# Stability of miRNA 5′terminal and seed regions is correlated with experimentally observed miRNA-mediated silencing efficacy

**DOI:** 10.1038/srep00996

**Published:** 2012-12-18

**Authors:** Naoki Hibio, Kimihiro Hino, Eigo Shimizu, Yoshiro Nagata, Kumiko Ui-Tei

**Affiliations:** 1 Department of Computational Biology, Graduate School of Frontier Sciences, University of Tokyo, 5-1-5 Kashiwanoha, Kashiwa-shi, Chiba-ken 277-8561, Japan; 2 Department of Biophysics and Biochemistry, Graduate School of Science, University of Tokyo, 7-3-1 Hongo, Bunkyo-ku, Tokyo 113-0033, Japan

## Abstract

MicroRNAs (miRNAs) are key regulators of sequence-specific gene silencing. However, crucial factors that determine the efficacy of miRNA-mediated target gene silencing are poorly understood. Here we mathematized base-pairing stability and showed that miRNAs with an unstable 5′ terminal duplex and stable seed-target duplex exhibit strong silencing activity. The results are consistent with the previous findings that an RNA strand with unstable 5′ terminal in miRNA duplex easily loads onto the RNA-induced silencing complex (RISC), and miRNA recognizes target mRNAs with seed-complementary sequences to direct posttranscriptional repression. Our results suggested that both the unwinding and target recognition processes of miRNAs could be proficiently controlled by the thermodynamics of base-pairing in protein-free condition. Interestingly, such thermodynamic parameters might be evolutionarily well adapted to the body temperatures of various species.

MicroRNAs (miRNAs) are a large family of single-stranded non-coding RNAs that direct the post-transcriptional repression of protein-coding genes in metazoans. In human cells, more than 1,500 miRNAs have been identified and are predicted to regulate the activity of numerous protein-coding genes to control many developmental and cellular processes including proliferation, apoptosis, and differentiation. Also, miRNAs are dysregulated in tumors and function as tumor suppressors or oncogenes. To explore the regulation of gene silencing mediated by miRNAs, it is necessary to identify the target genes and silencing efficacies of each miRNA. Recently, the miRNA target genes have been computationally predicted using algorithms that have been validated experimentally^1^,^2^,^3^. However, the mechanistic features that determine miRNA-mediated silencing efficacy remain poorly understood.

Primary miRNAs (pri-miRNAs) are expressed from the genome and processed by the double-stranded RNA cleavage enzyme Drosha in the nucleus to generate ~70-nucleotide (nt)-long precursor-miRNAs (pre-miRNAs)^4^,^5^,^6^. The pre-miRNAs are exported to the cytoplasm^7^,^8^ where they are further processed by the enzyme Dicer to generate ~22-nt miRNA duplexes^5^,^9^,^10^. The miRNA duplex is loaded into Argonaute (Ago) protein in the RNA-induced silencing complex (RISC) as a double strand^11^,^12^,^13^,^14^,^15^ and subsequently unwound into a single-strand in the RISC^14^,^15^. The retained strand acts as a guide to recruit the silencing complex mainly to the 3′ untranslated region (UTR) of target mRNA to promote translational repression^16^,^17^, with few exceptions^18^,^19^,^20^. In mammalian cells, another type of double-stranded small RNA, small interfering RNA (siRNA), is typically used as a tool for gene silencing in loss-of-function experiments and is expected to be applicable to gene therapy. The silencing mechanism of siRNA is very similar but not entirely consistent with that of miRNA. Usually, siRNAs are exogenously introduced into the cells as double-stranded RNAs ~21 nt in length with 2-nt 3′ overhangs. The passenger strands of most double-stranded siRNAs loaded onto RISC are cleaved by Ago2 protein, the catalytic component of RISC, and degraded^14^,^15^. The guide strand retained by RISC shares full sequence complementarity with its intended target gene and triggers enzymatic cleavage of mRNA by Ago2 protein between the nucleotides facing the siRNA guide strand, nucleotides 10 and 11, via RNA interference (RNAi)^21^,^22^,^23^,^24^,^25^,^26^. In addition, the guide strand recognizes many mRNAs with partial complementarity, mostly involving residues 2–8 from the 5′ termini (seed region), which are referred to as off-target effects. Accumulated evidence from large-scale knockdown experiments^2^,^3^,^27^,^28^,^29^,^30^,^31^,^32^,^33^ suggested that siRNA could generate off-target effects through a mechanism similar to that of target silencing by miRNA^2^,^3^,^32^. Thus, miRNA-mediated silencing and siRNA-based off-target effects may use similar machinery as downstream target recognition processes.

Previously, we found that the efficacies of seed-dependent off-target effects of siRNAs are strongly correlated with the calculated thermodynamic stabilities of seed-target duplexes^33^. However, unlike siRNA off-target effects, the efficacy of miRNA-mediated gene silencing was not simply correlated with seed-target duplex stability. Here, we demonstrated using mathematization of base-pairing stability that the efficacy of miRNA-mediated gene silencing was determined principally by the combinatorial thermodynamic parameters, which might reflect the easiness in unwinding in addition to the base-pairing stability in the seed-target duplex. Furthermore, because temperature is a key regulator of base-pairing stability, the thermodynamic properties of miRNAs of various species with different body/rearing temperatures were evaluated. Interestingly, we found that the thermodynamic stability between the miRNA seed region and target mRNA is well correlated to the body temperatures of various species.

## Results

### Variation in miRNA-mediated gene silencing activity

**Figure 1:**
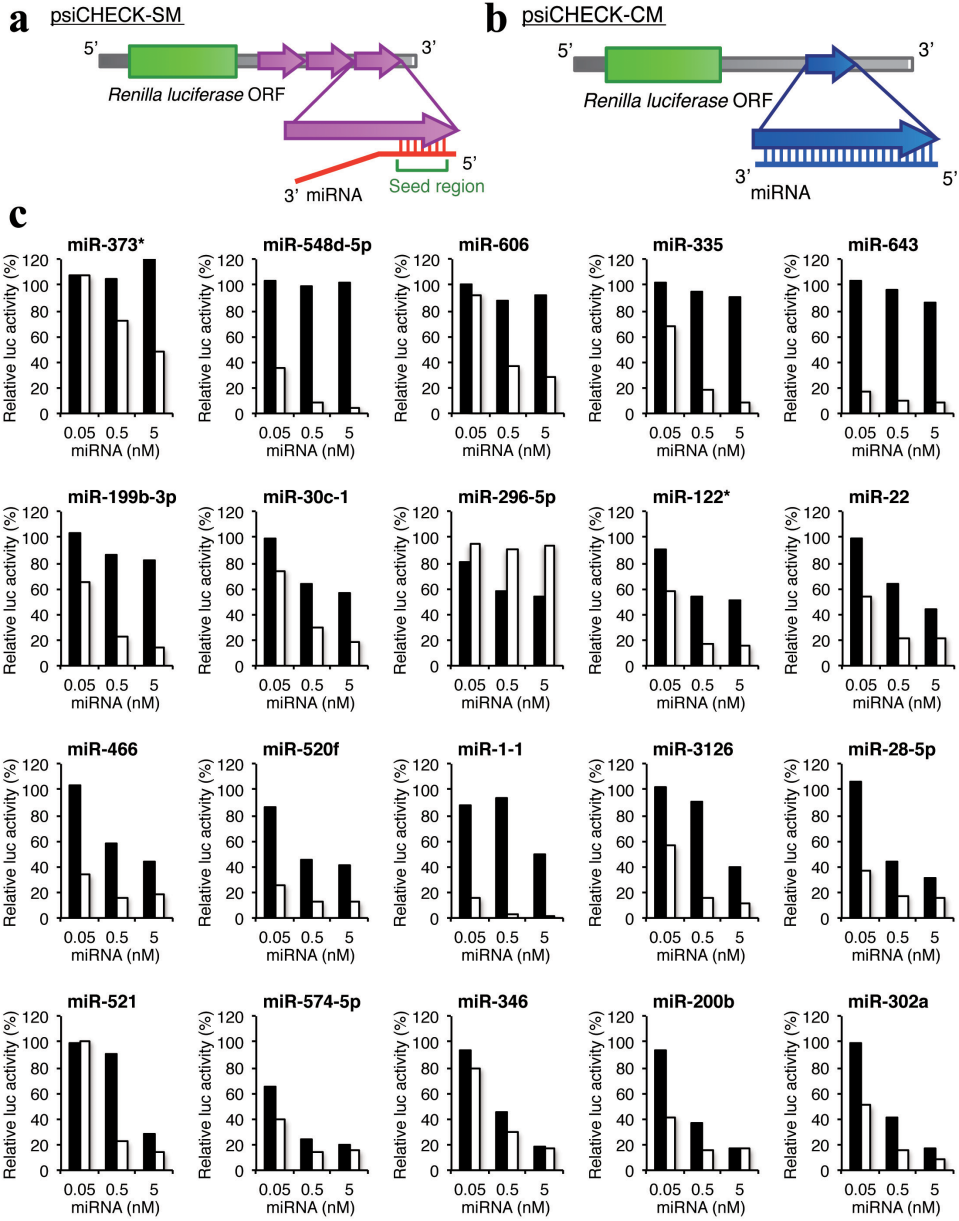
MiRNA-mediated gene-silencing assay using reporter plasmids. (a) Structure of psiCHECK-SM, which has three tandem repeats in the *Renilla* luciferase 3′ UTR. (b) Structure of psiCHECK-CM, which has a complete-matched target sequence in the *Renilla* luciferase 3′ UTR. (c) The miRNA-mediated gene-silencing activities in HeLa cells as a function of miRNA concentration at 0.05, 0.5, and 5 nM. The indicated concentration of synthetic miRNA, psiCHECK-SM construct (10 ng), and pGL3-control (100 ng) were simultaneously transfected into HeLa cells, and *Renilla* luciferase activity / firefly luciferase activity was determined 24 hours after transfection. The gene-silencing efficiencies of target mRNA varied significantly depending on the miRNA duplex used for transfection. The sequences of miRNAs and target mRNAs are shown in Figure S1. The ordinate represents relative luciferase activity (%) and the abscissa represents miRNA concentration. The black bar indicates the result of psiCHECK-SM. The white bar, the result of psiCHECK-CM.

To determine the relationship between miRNA structures/sequences and their direct silencing efficacies, we performed reporter assays. Three tandem repeats of partially complementary sequences containing a seed-matched sequence of each of 20 arbitrarily chosen human miRNAs (Figure S1) were introduced into the *Renilla* luciferase 3′ UTR in the psiCHECK plasmid, hereafter called psiCHECK-SM (Figure 1a). The pGL3-Control, encoding firefly luciferase, and each psiCHECK-SM construct were transfected into human HeLa cells along with the corresponding miRNA. Twenty-four hours after transfection, the relative luciferase activity was measured as a function of miRNA concentration (Figure 1c). Little or no silencing effects were observed by transfection with any of the 20 miRNAs at 0.05 nM, and six miRNAs at 0.5 nM reduced the luciferase activity to below 50%. For six miRNAs (miR-373*, miR-548d-5p, miR-606, miR-335, miR-643, and miR-199b-3p), no appreciable silencing effects were seen even when the miRNA concentration was increased to 5 nM. We also performed the reporter assay mimicking the RNAi effect using psiCHECK-CM, which has a complete-matched sequence of each miRNA in the *Renilla* luciferase 3′ UTR (Figure 1b). Previously, we reported that siRNAs can be divided into three groups; namely, class I: highly functional, class II: intermediate, and class III: ineffective siRNAs, or siRNAs with long GC stretches (>9) have little silencing activity^34^. Of the 20 miRNAs, 15 miRNAs were classified into class I and five into class II. Excluding miR-296-5p, all of the miRNAs strongly reduced the relative luciferase activities in the psiCHECK-CM reporter assay to less than 50% at 0.5 nM (Figure 1c). The same RISC may play a role in RNAi and miRNA-mediated gene silencing^35^. Thus, part of the miRNAs may have little or no gene silencing activity on the partially complementary target mRNAs, even when engaged by the RISC. In contrast, miR-296-5p reduced the luciferase activity of psiCHECK-SM, but not psiCHECK-CM. miR-296-5p is classified into class II and has a 10-nt GC stretch. Thus, miR-296-5p can be held on the RISC for miRNA-mediated gene silencing, but could not repress the complete-matched target, likely due to the long GC-stretch by which the cleaved target might not be easily released from the RISC. In a previous study, we reported that the content of luciferase mRNA produced within cells was about 300 copies/ng total RNA (one-hundredth that of β-actin mRNA) under our experimental conditions, and that the luciferase activities measured using different psiCHECK-SM constructs were almost proportional to the levels of mRNA^33^. Thus, under our conditions, the majority of the luciferase activity reduction was attributable to miRNA-mediated luciferase mRNA degradation.

### Correlation between miRNA silencing and combined stability

**Figure 2:**
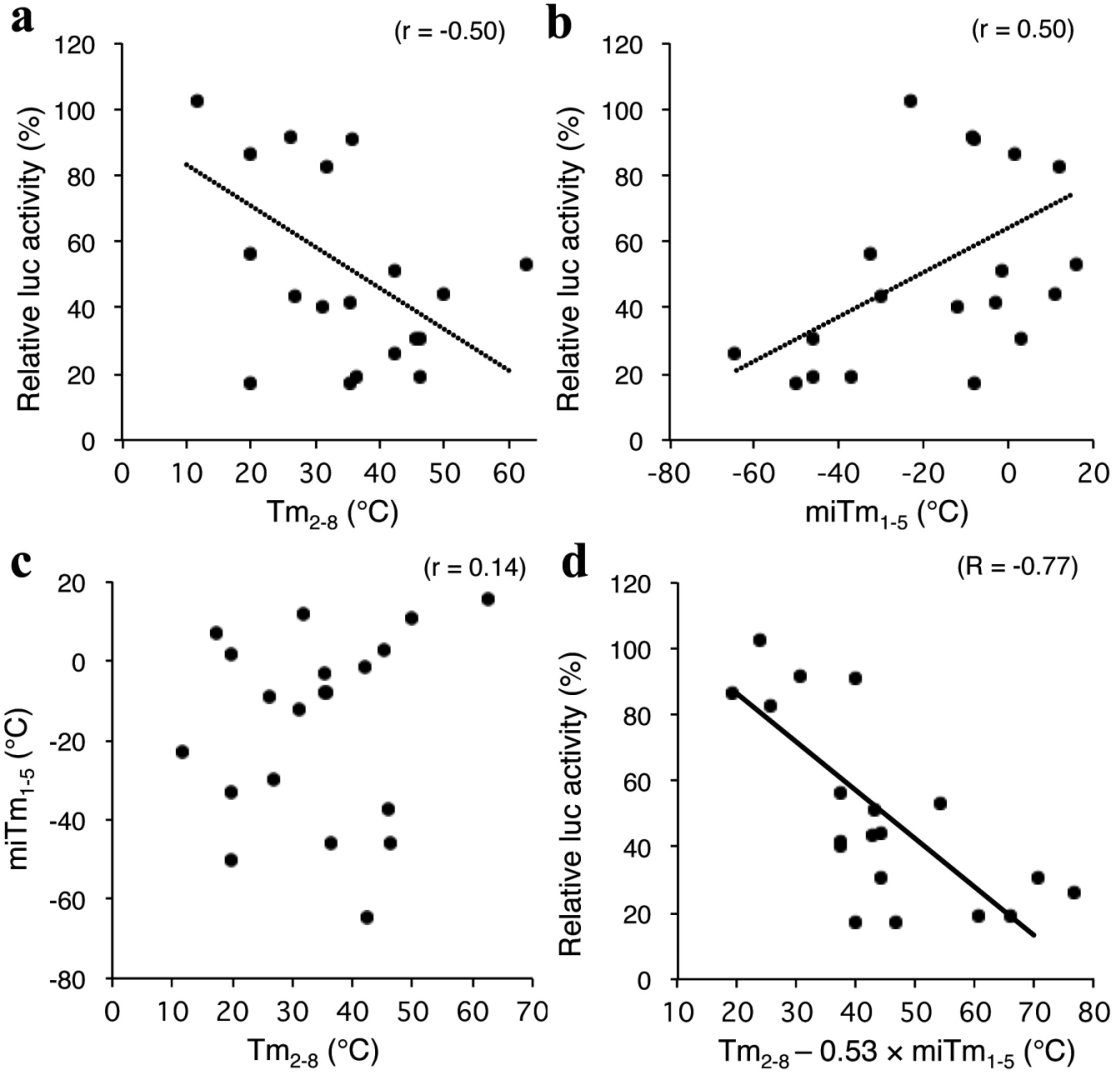
Close relationship between efficacy of miRNA-mediated gene silencing and combinatorial thermodynamic stabilities in the seed-target duplex and the 5′ terminal miRNA duplex. (a) Correlation between miRNA-mediated gene-silencing activity (relative luciferase activity) and the calculated Tm_2–8_ of the protein-free seed duplex. The set of luciferase activities compromised by miRNA-mediated gene silencing at 5 nM miRNA concentration was obtained from Figure 1. Tm_2–8_ value of the protein-free seed region was calculated using the nearest neighbor method. Relative luciferase activity and calculated Tm_2–8_ showed little, if any, correlation with each other and had a coefficient of −0.50 (p ≤ 0.05). (b) The luciferase activity compromised by miRNA-mediated gene silencing at 5 nM miRNA concentration showed little, if any, correlation with calculated miTm_1–5_ (r = 0.50, p ≤ 0.05). (d) Correlation between the calculated Tm values in the seed region (positions 2–8) and 5′ terminal region (positions 1–5). The calculated Tm_2–8_ and the calculated miTm_1–5_ showed no correlation with each other and had a coefficient of 0.14 (p > 0.05). (d) Correlation between miRNA-mediated gene-silencing activity and the calculated Tm_2–8_ − 0.53 × miTm_1–5_ value. Relative luciferase activity and calculated Tm_2–8_ − 0.53 × miTm_1–5_ were highly negatively correlated with each other and had a coefficient of −0.79 (p ≤ 0.01).

We previously demonstrated the seed-dependent off-target effects of siRNA measured using a luciferase reporter assay at a concentration of 50 nM, which was negatively correlated with thermodynamic stability in the duplex formed between the seed region of the siRNA guide strand and its target mRNA with a correlation coefficient (r) of −0.72^33^. Hence, because the siRNA seed region is a primary target-recognition region, it is possible that the highly stable seed-target duplex results in a strong siRNA seed-dependent off-target effect. This correlation was successfully calculated when melting temperature (Tm) was used as a measure of duplex thermodynamic stability. The off-target effect of siRNA is generated through a mechanism similar to that of target silencing by miRNA^2^,^3^,^32^. Unlike the siRNA, the efficacy of miRNA-mediated luciferase gene silencing using psiCHECK-SM was poorly correlated (r = −0.50) with the calculated Tm values in the seed (positions 2–8)-target duplex (Tm_2–8_) at 100 mM NaCl (Figure 2a).

**Figure 3:**
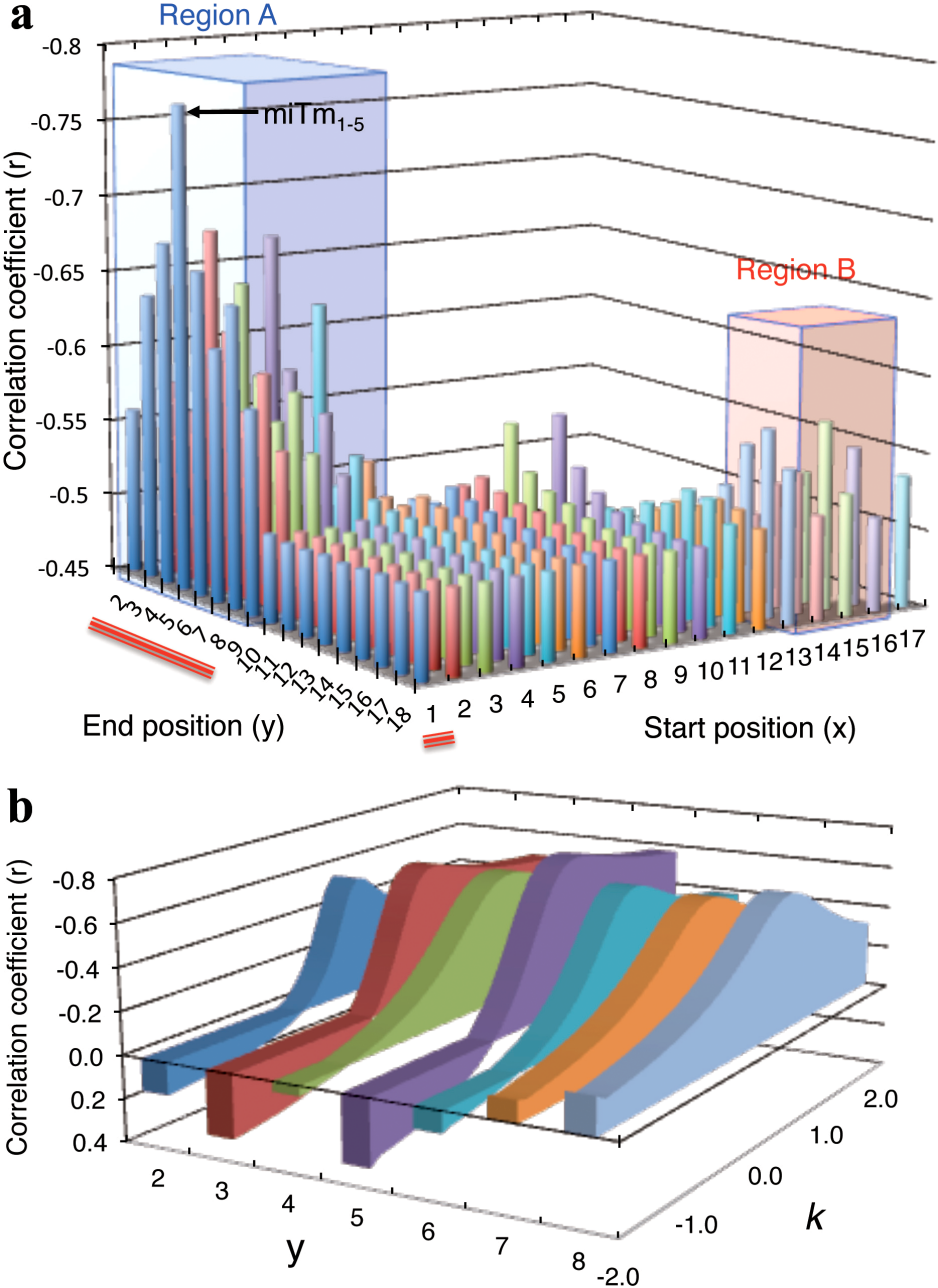
Optimization of the regions and multiplicative factor to estimate possible miRNA-mediated silencing efficacy. (a) Determination of the optimal start position “x” and end position “y” in the formula Tm_2–8_ − *k* × miTm_x–y_, which shows the highest correlation coefficient with the efficacy of miRNA-mediated gene silencing (relative luciferase activity), using the most-to-least optimal regions. The optimal multiplicative factor *k*, which was determined as shown in Figure 3b, was used to calculate Tm_2–8_ − *k* × miTm_x–y_ for each region. Red lines indicate the region represented in Figure 3b. Regions A and B are areas of high correlation with relative luciferase activity. The strongest correlation was observed in the region 1–5. (b) Examples of fluctuating correlation coefficients between silencing efficacies and values represented as Tm_2–8_ − *k* × miTm_x–y_ (x was fixed as 1, 2 ≤ y ≤ 8) depending on *k* value (−2 ≤ *k* ≤ 2). The prominent peaks of the optimal correlation coefficients of Tm_2–8_ − *k* × miTm_x–y_ with silencing efficiency were obtained around *k* values of 0.5. The strongest correlation was obtained at *k* value of 0.53 in the region from x = 1 to y = 5.

We then considered that miRNA-specific features that are involved in the silencing process before target recognition may be responsible for the efficacy of miRNA-mediated silencing. miRNA has specific structural features such as an internal bulge or mismatch (see Figures 7 and S1), but siRNA is simply composed of perfectly complementary double-stranded RNAs. We examined the involvement of thermodynamic stability in the miRNA duplex (miTm) by calculating Tm values considering the internal bulge/mismatch. To determine the optimal region with a high correlation with silencing efficacy, miTm values in the most-to-least optimal regions in the miRNA duplex were calculated. Each of these values was subtracted from the Tm_2–8_ value and their correlation coefficient was estimated with respect to silencing efficacy (luciferase activity) according to the formula Tm_2–8_ − *k* × miTm_x–y_, where x is the start nucleotide position (1 ≤ x ≤ 17), y is the end nucleotide position (2 ≤ x ≤ 18), and *k* is a multiplicative factor (Figure 3). Surprisingly, the stability in the 5′ terminal region had a significant effect on silencing efficacy. When the optimal *k* value was used for each region, the Tm_2–8_ − *k* × miTm_x–y_ values in region A shown in Figure 3a starting from position x = 1~5 and ending at position y = 2~9 were closely correlated with silencing efficacy (r = −0.51 to −0.77). The strongest negative correlation coefficient (−0.77) was obtained when miTm_1–5_ was used; the resultant formula was Tm_2–8_ − *k* × miTm_1–5_ (Figures 2d and 3a). Weak but significant correlations were also observed in region B from nucleotides 13~16 to 16~17 (r = −0.53 to −0.57). The regions from nucleotide 1~12 ending at 10~18 showed little or no correlation. The optimal *k* values were independently calculated for each region, and the results at positions x = 1 to y = 2~8 are shown in Figure 3b. The prominent peaks of correlation coefficients were obtained around 0.5. The closest relationship was found when *k* was 0.53. miTm_1–5_ alone showed little correlation (r = 0.50) with silencing efficacy (Figure 2b), as in the case with Tm_2–8_ alone (r = −0.50) (Figure 2a). There was no correlation between the values of miTm_1–5_ and those of Tm_2–8_ (r = 0.14), although these regions partially overlapped, indicating that the 5′ terminal structures of miRNAs are extraordinarily diversified independent of their nucleotide sequence (Figure 2c). Thus, the silencing efficacy might be estimated based on the combinatorial parameters representing the stability of the miRNA 5′ terminal duplex, miTm_1–5_, and base-pairing stability between the seed region and target mRNA, miTm_2–8_. The following formula should appropriately predict miRNA-mediated gene-silencing efficacy: Tm_2–8_ − 0.53 × miTm_1–5_.

A considerable deviation was also observed in luciferase activity measurements (Figure 2d). This may have been due in part to differences in the non-seed sequence and/or its counterpart in the target mRNA (see Figure S1, right column) because the target sequences that correspond to the non-seed region make an appreciable contribution to target recognition by miRNAs and/or siRNAs in microarray profiling^2^,^3^,^28^.

### Different silencing efficacies of miRNAs with common seed sequences or common nucleotide compositions

**Figure 4:**
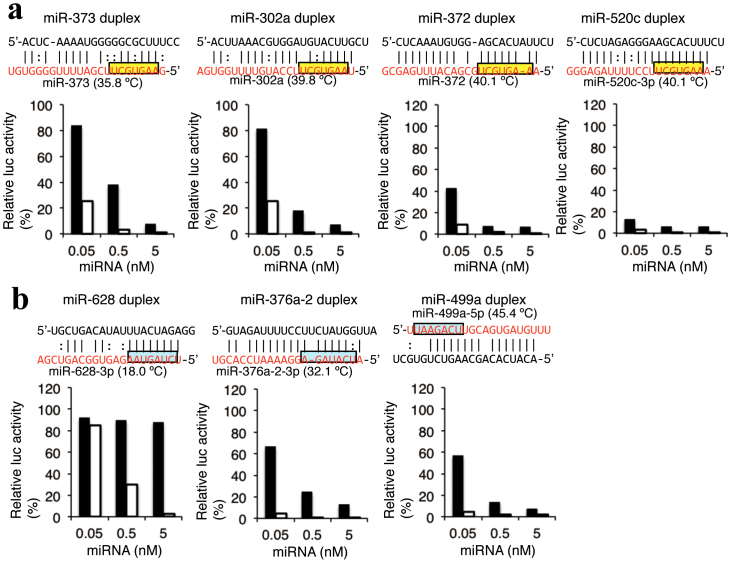
Tm_2–8_ − 0.53 × miTm_x–y_ value-dependent different silencing activities of miRNAs. The indicated concentration of synthetic miRNA, psiCHECK-SM construct (10 ng), and pGL3-control (1 µg) were simultaneously transfected into HeLa cells, and *Renilla* luciferase activity / firefly luciferase activity was determined 24 after transfection. The results of luciferase reporter assays using (a) miR-302a-3p/372/373-3p/520c-3p family miRNAs with same seed sequences but different structures, or (b) miR-376a-2-3p/499-5p/628-3p with same composition of nucleotides in the seed regions. The value of Tm_2–8_ − 0.53 × miTm_1–5_ of each miRNA was represented in parenthesis. The structures of psiCHECK-SM and psiCHECK-CM reporters are same as those shown in Figure 1a and 1b. The black bar indicates the result of psiCHECK-SM. The white bar, the result of psiCHECK-CM. The ordinate represents relative luciferase activity (%) and the abscissa represents miRNA concentration. Yellow indicates the common seed sequence in miR-302a-3p/372/373-3p/520c-3p family miRNAs, blue indicates the seed region with same nucleotide compositions in miR-376a-2-3p/499-5p/628-3p miRNAs.

Although miRNAs recognize their target genes based on the complementarity to the seed region, our results suggested that miRNAs with the same seed sequences but different duplex structures may have different silencing efficacies according to the Tm_2–8_ − 0.53 × miTm_1–5 _values. To evaluate this possibility, we investigated the silencing activities of miR-302a/372/373/520c-family miRNAs containing a common seed sequence (AAGUGCU), so their Tm_2–8_ values are identical. Members of this miRNA family are known to induce miRNA-induced pluripotent stem (mirPS) cells^36^, and we have reported that the expression levels of many genes with seed complementary sequences in their 3′UTRs are commonly regulated^20^. As shown in Figure 4a, miR-372 and miR-520c-3p have the same Tm_2–8_ − 0.53 ×miTm_1–5 _values of 40.1°C, but the values of miR-373 and miR-302a are 35.8°C and 39.8°C, respectively. The reduction of luciferase activity of psiCHECK-SM was weak (38% of relative luc activity at 0.5 nM miRNA duplex) after treatment with miR-373, while rather strong activity (18%) was induced by the miR-302a duplex. Furthermore, miR-372 and miR-520c duplexes significantly reduced the luciferase activities of psiCHECK-SM to 8% and 6%, respectively. The results indicated that their silencing efficacies were correlated with their Tm_2–8_ − 0.53 × miTm_1–5 _values. We also tried to evaluate the efficiencies of the other miRNAs including miR-1302-1/1302-2/1302-7/1302-8, miR-7-1/7-2/7-3, and artificially mutated miR-30c-1s and miR-643s, which have identical seed sequences but different duplex structures. However, we could not perform the experiments since part of these miRNAs were not successfully annealed, leading to failed miRNA duplex formation, under our annealing conditions (see Methods).

Furthermore, we evaluated the silencing efficiencies of miRNAs with the same seven nucleotide compositions in their seed regions (A = 3, U = 2, G = 1, C = 1), but in different orders (Figure 4b). The Tm_2–8_ − 0.53 × miTm_1–5 _values of miR-628-3p, miR-376a-2-3p, and miR-499a-5p were 18.0, 32.1, and 45.4°C, respectively. Their relative luciferase activities were 89, 20, and 14% at 0.5 nM miRNA duplex, respectively, representing a good agreement with their Tm_2–8_ − 0.53 ×miTm_1–5 _values.

### Thermodynamic properties of miRNAs in various species

**Figure 5:**
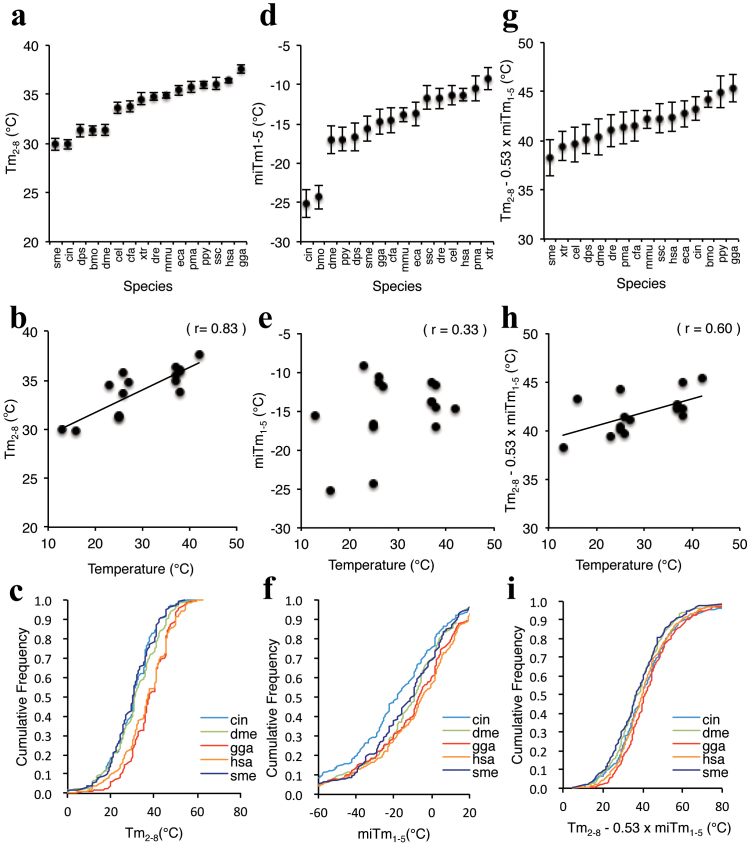
Thermodynamic profiles of miRNAs in different organisms. Comparison of thermodynamic profiles of miRNAs in various organisms registered in miRBase. Thermodynamic parameters are shown in table S1. MiRNAs registered as double strands with 2-nt 3′ overhangs in both strands of 16 different organisms were ordered as a function of average Tm_2–8_ (a), miTm_1–5_ (d), and Tm_2–8_ − 0.53 × miTm_1–5_ (g). The correlation between temperature and Tm_2–8_ (b), miTm1–5 (e), and Tm_2–8_ − 0.53 × miTm_1–5_ (h). The miRNAs for *C. elegans, C. intestinalis, D. melanogaster, G. gallus, H. sapiens*, and *S. mediterranea* were ordered as a function of calculated Tm_2–8_ (c), miTm_1–5_ (f), and Tm_2–8_ − 0.53 × miTm_1–5_ (i). Temperatures imply body temperatures for chickens (*gga*), humans (*hsa*), mice (*mma*), dogs (*cfa*), horses (*eca*), orangutans (*ppy*), and pigs (*ssc*), and rearing temperatures for planarians (*sme*), ascidians (*cin*), African frogs (*xtr*), nematodes (*cel*), silkworms (*bmo*), *D. melanogaster* (*dme*), *D. pseudoobscure* (*dps*), lampreys (*pma*), and zebrafish (*dre*). The three-letter abbreviations are provided in figures. Results of a two-sided K-S test for miRNA-mediated gene-silencing activities are as follows: MiRNA Tm_2–8_ values of H. sapiens compared to those of *C. elegans*, P ≤ 3.1 × 10^−7^; *C. intestinalis*, P ≤ 2.2 × 10^−16^; *D. melanogaster*, P ≤ 2.2 × 10^−13^; *G. gallus*, P ≤ 3.7 × 10^−2^; and *S. mediterranea*, P ≤ 2.2 × 10^−16^. MiRNA miTm_1–5_ values of H. sapiens compared to those of *C. elegans*, P ≤ 8.2 × 10^−4^; *C. intestinalis*, P ≤ 2.2 ×10^−16^; *D. melanogaster*, P ≤ 4.9 × 10^−6^; *G. gallus*, P ≤ 3.0 × 10^−2^; and *S. mediterranea*, P ≤ 2.0 × 10^−5^. MiRNA Tm_2–8_ − 0.53 × miTm_1–5_ values of *H. sapiens* compared to those of *C. elegans*, P ≤ 2.1 × 10^−2^; *C. intestinalis*, P ≤ 5.5 × 10^−1^; *D. melanogaster*, P ≤ 8.7 × 10^−7^; *G. gallus*, P ≤ 5.7 × 10^−3^; and *S. mediterranea*, P ≤ 1.8 × 10^−3^.

Small RNA-mediated gene silencing is a conserved phenomenon in metazoans^37^. In this study, we found that the efficacy of miRNA-mediated silencing could be successfully determined based on the thermodynamic properties of protein-free RNA duplexes. Thermodynamic propensity is naturally controlled by ambient temperature. Because the systemic or rearing temperatures of each species differs, the actual stability of the RNA duplex should differ by species. Thus, it is possible that functional miRNAs in each species are evolutionarily adapted according to temperature. We analyzed the thermodynamic parameters Tm_2–8_, miTm_1–5_, and Tm_2–8_ − 0.53 ×miTm_1–5_ using miRNAs of 16 different species registered in miRBase (Table S1). The average Tm_2–8_ values varied widely, from 29.9 to 37.6°C (Figure 5a). The planarian *Schmidtea mditerranea* and ascidian *Ciona intestinalis* are heterothermic animals that are usually maintained at approximately 10–15°C. The African frog *Xenopus tropicalis*, nematode *Caenorhabditis elegans*, silkworm *Bombyx mori*, *Drosophila melanogaster*, *Drosophila pseudoobscure*, lamprey *Petromyzon marinus*, and zebrafish *Danio rerio* are reared at 23–27°C. The body temperature of homothermic animals such as human *Homo sapiens*, mouse *Mus musculus*, dog *Canis familiaris*, horse *Equus caballus*, orangutan *Pongo pygmaeus*, and pig *Sus scrofa* is about 37°C. The body temperature of the chicken *Gallus gallus* is highest at about 42°C. In the present study, low-temperature animals preserved miRNAs with significantly low average Tm_2–8_ values (Table S1 and Figure 5b). In contrast, the miRNAs of high-temperature animals had high average Tm_2–8_ values. The cumulative fraction of the Tm_2–8_ values of the miRNAs of *S. mediterranea*, *C. intestinalis*, *D. melanogaster*, *H. sapiens,* and *G. gallus* clearly varied according to their temperatures (Figure 5c). An apparent correlation (r = 0.83) between Tm_2–8_ values and temperature was observed (Figure 5b), suggesting that miRNA sequences in the seed region are evolutionarily adapted to temperature.

We further calculated the average miTm_1–5_ value for the miRNAs of each species. The values varied significantly from −25.2 to −9.2°C (Table S1 and Figure 5d). The average miTm_1–5_ values of *B. mori* (−24.3°C) and *C. intestinalis* (−25.2°C) were significantly lower than those of the others (Table S1 and Figure 5e). The cumulative fractions of miTm_1–5_ also varied (Figure 5f), but were not correlated with temperature (r = 0.33; Figure 5e), suggesting that evolutionary pressure on the miRNA 5′ terminal regions is weak.

The thermodynamic parameters, Tm_2–8_ − 0.53 × miTm_1–5_ values, of the miRNAs of 16 species were evaluated (Figure 5g). Similar but not concordant results from Tm_2–8_ were obtained. The averaged Tm_2–8_ − 0.53 × miTm_1–5_ values varied from 38.2 to 45.4°C (Table S1 and Figure 5g). However, the cumulative fractions of Tm_2–8_ − 0.53 × miTm_1–5_ of miRNAs varied significantly according to species (Figure 5i) and the combinatorial stability of the seed-target duplex and the 5′ terminal region duplex correlated with temperature (r = 0.60; Figure 5h).

**Figure 6:**
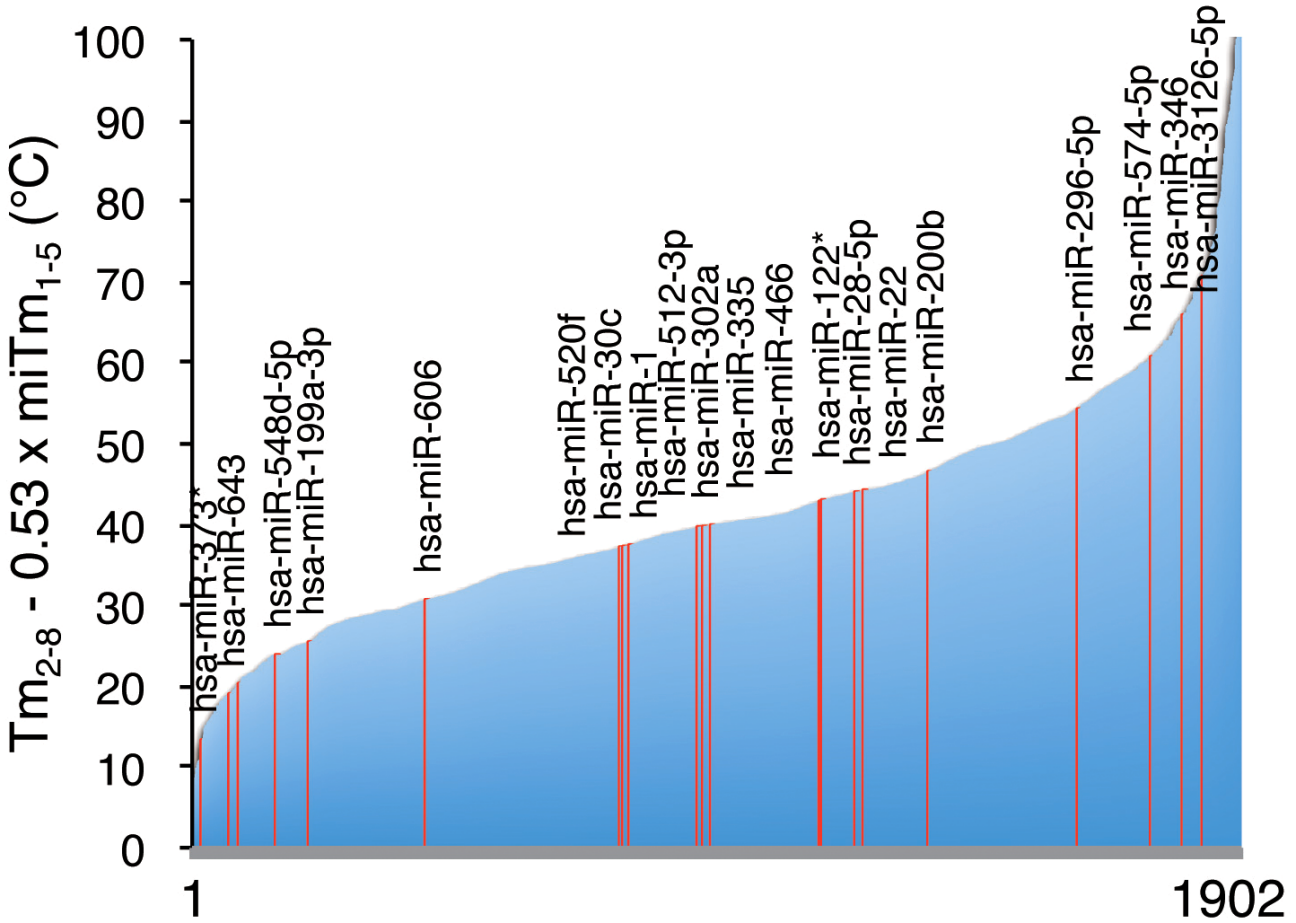
Predicted silencing efficiencies of a total of 1,830 human miRNAs registered in miRBase. The Tm_2–8_ − 0.53 × miTm_1–5_ values were ordered according to their values. The red lines indicate the miRNAs whose silencing effects were measures by reporter assays as shown in Figure 1.

We calculated the predicted miRNA-mediated silencing efficacy using the formula Tm_2–8_ − 0.53 × miTm_1–5_ for 1,902 human miRNAs registered in miRBase (Figure 6 and Table S2). The values ranged from 4.6°C to 203.8°C, suggesting that human miRNAs have enormously divergent silencing efficacies.

## Discussion

**Figure 7:**
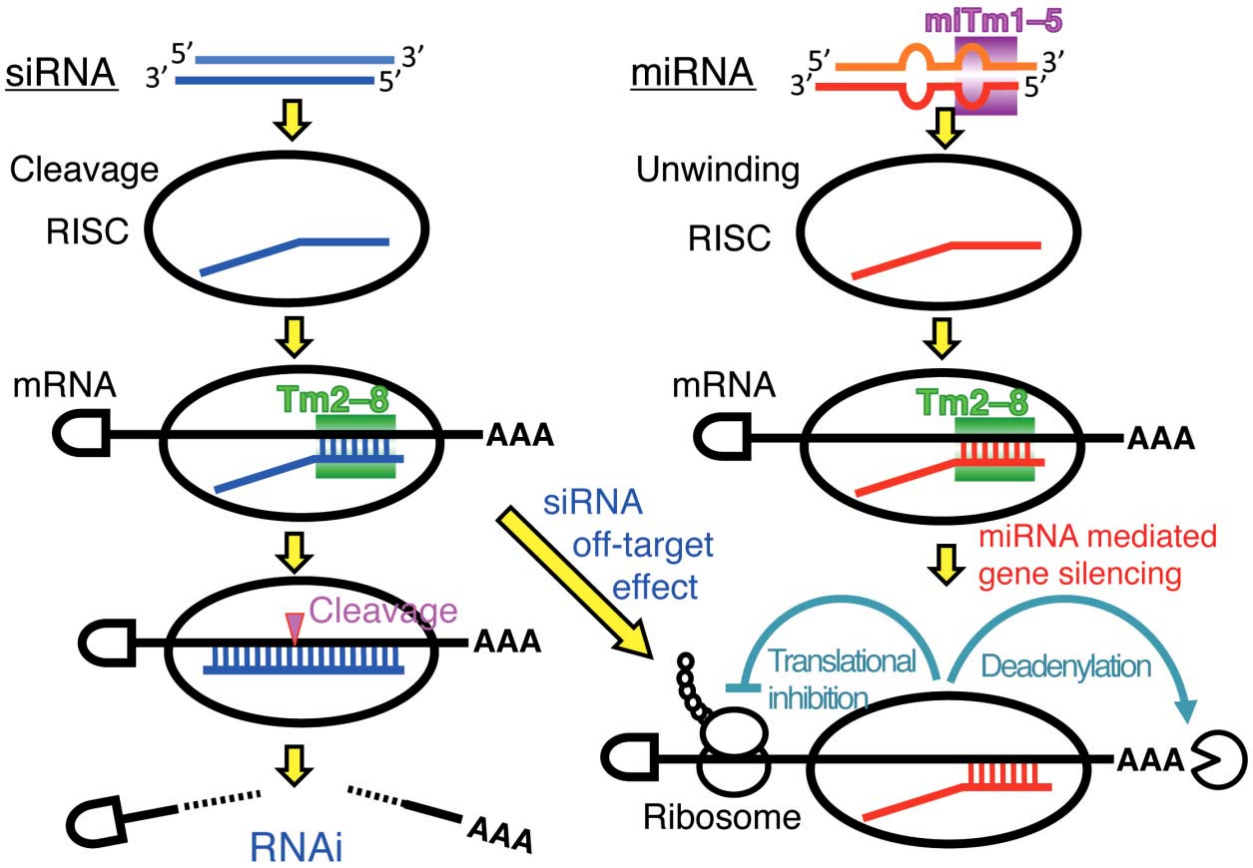
Possible thermodynamic control of miRNA/siRNA-mediated gene-silencing activity. Left and right columns indicate the siRNA-based off-target pathway and miRNA-mediated silencing pathway, respectively. The miRNA/siRNA duplex is unwound to a single-stranded RNA, which loads onto the RISC, and acts as a guide to recruit the silencing complex to the 3′ UTR of the target mRNA to promote translational repression or cleavage. In the siRNA pathway, the efficiencies of the siRNA seed-dependent off-target effects were strongly correlated with the calculated thermodynamic stabilities in the seed-target duplexes, Tm_2–8_. In the miRNA pathway, the efficacies of miRNA-mediated silencing are determined by the combined thermodynamic parameters that might reflect their unwinding properties (miTm_1–5_) in addition to their base-pairing stabilities in the seed-target duplex (Tm_2–8_).

The efficacy of miRNA-mediated gene silencing was estimated based on the combinatorial thermodynamic parameters, Tm_2–8_ − 0.53 × miTm_1–5_, of protein-free RNA duplexes in the regions administrating unwinding efficacy (miTm_1–5_) and base-pairing stability with target mRNA (Tm_2–8_), as shown in Figure 7. Our results are in excellent agreement with the known silencing machineries. First, an RNA strand containing the thermodynamically less stable 5′ end is preferentially entrapped on the RISC^33^,^38^,^39^. The internal bulge/mismatch is thought to form a less stable base-pairing, suggesting that miRNAs with such relaxed structures on the 5′ end are easily retained by RISC. Second, miRNA recognizes target mRNA with seed-complementary sequences^2^,^3^,^32^. The stability between seed region and target mRNA is a determinant of the efficacy of siRNA off-target effects^33^. Thus, the high stability of the seed-target duplex might function as a positive factor, but that in the miRNA duplex the 5′ end might be a negative regulator of target gene silencing. Although these regions overlap, the stability of the duplex formed between the miRNA seed and target mRNA is defined by the nucleotide sequence, while the stability within the miRNA duplex is largely attributable to structural features rather than the nucleotide sequence. Thus, our results suggest that these two regions may coordinately but independently regulate the silencing machinery. Furthermore, the coefficient factor, 0.53, in the mathematical formula suggested that the contribution of the nucleotides 1–5 in miRNA for the silencing efficacy might be about half of that of nucleotides 2–8 in the seed region.

Seed pairing is known to be both necessary and sufficient for target regulation by microRNAs in some experimental contexts^40^. We performed experiments focused on the canonical base pairing in the seed region, and presented a model of predicting miRNA silencing efficacy. However, alternative modes of target recognition by miRNAs have been reported recently, including 3′-compensatory sites^3^, centered sites^41^, or bulged sites^42^,^43^. In our experiments, it was also apparent that region B, corresponding to the 3′-compensatory sites from nucleotides 13~16 to 16~17, contributed to the silencing efficacy to some extent (Figure 3a). The nucleotides from 13~16 are required for increased efficacy but are only slightly effective compared to those without the supplementary pairing^3^; they play a modest role in target recognition. Furthermore, this site can compensate for a single-nucleotide bulge/mismatch in the seed region. We attempted to incorporate the miTm_13–16_ values into the thermodynamic parameters, but no significant improvement was observed (data not shown), probably because only target mRNAs with seed-complementary sequences without internal bulges were used in our luciferase reporter assays. It was also reported that miRNA represses the expression of mRNAs with seed-complementary sequences with bulges in the mRNA side^43^. Although we did not examine the effects on the bulged targets in this study, the Tm_2–8_ value in seed-target duplex is identical to that in the duplex formed between miRNA and a non-bulged target, suggesting that our model is applicable. However, Ha *et al.*^42^ reported that miRNA also represses mRNAs when bulges are formed in the miRNA side of the seed-target duplex. In most of these cases, the Tm_2–8_ values should change according to the bulged structures, possibly leading to different silencing efficacies. Furthermore, miRNAs that lack both perfect seed pairing and 3′-compensatory pairing and instead have 11–12 contiguous Watson-Crick pairs in the center of the miRNA are also functional as miRNA cleavage substrates *in vitro*^41^. In addition, a given miRNA was shown to generate non-canonical functioning heteroduplexes with targets that do not contain the miRNA seed by molecular dynamics analyses, indicating that the spectrum of potential targets for a miRNA includes a wide-spectrum of seed-less targets and thus substantially differs from what is anticipated based on the canonical seed mode^44^. However, we did not consider centered sites or non-canonical seed-less targets in this study.

We previously reported that the efficiency of siRNA seed-dependent off-target silencing is strongly correlated with Tm_2–8_ values^33^. Since siRNA forms perfectly complementary double-stranded RNA, the Tm values at positions 1 to 5 (Tm_1–5_) showed a strong positive correlation with the reduction in luciferase activity (r = 0.61; Figure S2b) similar to Tm_2–8_ (r = 0.80), as might be expected (Figure S2c). Thus, Tm_1–5_ might not be a good parameter for estimating the strand separation (unwinding) efficiency of siRNA. The efficacy of the siRNA off-target effect was not correlated with Tm_2–8_ − 0.53 × Tm_1–5_ (r = 0.47; Figure S2d). A possible explanation is that the region responsible for unwinding in the siRNA duplex differs from region 1–5 in miRNA. To determine the optimal region for the siRNA off-target effect, Tm values in the most-to-least optimal regions in siRNA duplexes were calculated in the same manner as for miRNA (Figure S3a). However, non-significant improvement in the efficiency of the siRNA off-target effect was observed when the optimal values (x = 5, y = 14, and *k* = 1.0) were used in the formula Tm_2–8_ − *k* × Tm_x–y_. Furthermore, no prominent peak in *k* value was detected for any position (Figure S3b), suggesting that the efficiency of the siRNA off-target effect is determined primarily by the stability of the seed-target duplex, as reported previously^33^. For siRNA-based off-target effects, the siRNA guide strand in the RISC cleaves the passenger strand with the Ago2 protein leading to its dissociation from RISC^11^,^12^,^45^. However, because most miRNA duplexes contain bulges to prevent cleavage, the miRNA* strand dissociates by unwinding^14^,^15^,^46^. Thus, instability in the 5′ terminal region might be essential for unwinding of the miRNA duplex, but might not be necessary for cleavage of the siRNA passenger strand.

The thermodynamic properties of miRNAs vary significantly among organisms (Figure 5). One causal factor of this may be natural selection in response to differences in temperature (body or rearing temperature). Considering the chemical characterization of nucleic acids, an RNA duplex with low thermodynamic stability would not be formed in the cells of an organism with a high temperature. The Tm_2–8_ and Tm_2–8_ − 0.53 × miTm_1–5_ values of the miRNAs of 16 different species were strongly correlated with the temperature of each species (Figure 5c and 5i), indicating that organisms with higher and lower temperatures possess miRNAs with higher and lower seed-target duplex stabilities, respectively. This suggests that the stabilities in the miRNA seed sequences are evolutionarily selected according to the adaptive temperature of each organism. In contrast, the diversity of miTm_1–5_ values was marginally affected by temperature (Figure 5f). However, since the values varied significantly, positions 1–5 may produce variation of silencing efficiencies independent of temperature. Thus, the functions of miRNA 5′ terminal and seed regions in miRNA-mediated gene silencing may differ. Some miRNAs, such as let-7^47^,^48^, miR-34^49^, miR-124^50^, and miR-125^49^, are known to be evolutionarily conserved and their target sites are conserved among various species^51^. Furthermore, the conserved miRNAs are ancient animal miRNAs whose localization in tissues are closely coupled in evolution^52^. The conserved miRNAs may be functional across species if their Tm_2–8_ values are high. The Tm_2–8_ values of let-7, miR-34, miR-124, and miR-125 were high at 37.6, 45.5, 40.8, and 45.2°C, respectively, suggesting that these conserved miRNAs play similar roles due to their high stability in seed-target duplexes.

## Methods

### miRNA synthesis

MiRNA duplexes were essentially chemically synthesized (Sigma) in accord with the sequences registered in the miRBase^53^ to mimic the structure of endogenous miRNAs. MiRNAs used in Figure 4 were synthesized as single-stranded RNAs, and annealed to form miRNA duplexes. For annealing, both strands of miRNA duplexes were mixed at 25 µM in 10 mM Tris-HCl (pH8.0) with 20 or 100 mM NaCl for 5 min at 95°C, and gradually decreased to room temperature. The duplex formation was verified using 15% polyacrylamide gel electrophoresis in 0.5 x TBE. The sequence of the synthetic miRNAs (miR-1-1, miR-22, miR-28, miR-30c-1, miR-122, miR-199b, miR-200b, miR-296, miR-302a, miR-335, miR-346, miR-373, miR-466, miR-520f, miR-521, miR-548d, miR-574, miR606, miR-643, miR-3126, siGY441) and their duplex structures are shown in Figure S1 (left column), and other miRNA sequences/structures are shown in Figure 4. siGY441 possesses unrelated sequence with *Renilla* luciferase gene, and used as negative control.

### Construction of seed-matched luciferase reporters

All of the reporter plasmids constructed were derivatives of psiCHECK-1 (Promega). Oligonucleotides with three tandem repeats of target sequence complementary to each miRNA (miR-1-1, miR-22, miR-28, miR-30c-1, miR-122, miR-199b, miR-200b, miR-296, miR-302a, miR-335, miR-346, miR-373, miR-466, miR-520f, miR-521, miR-548d, miR-574, miR606, miR-643, miR-3126) seed sequence were chemically synthesized with cohesive XhoI/EcoRI ends (table S3). They were then inserted into the corresponding restriction enzyme sites in *Renilla* luciferase 3′ UTR of psiCHECK-1 to generate psiCHECK-SM. Oligonucleotides possessing fully complementary sequence to each miRNA was chemically synthesized with cohesive XhoI/EcoRI ends (table S4) and inserted into psiCHECK-1 to generate psiCHECK-CM. The construct was purified with Genopure Plasmid Midi Kit (Roche), and sequences of insert regions were ascertained. Each of the inserted targets was expressed as part of the 3′ UTR region of *Renilla* luciferase mRNA in transfected cells.

### Cell culture and transfection

Human HeLa cells were cultured at 37°C in Dulbecco's Modified Eagle's medium (DMEM, Invitrogen) supplemented with 10% heat inactivated fetal bovine serum (FBS, Sigma). The cells were plated on 24- or 96-well culture plates (1.0 × 10^5^ cells ml^−1^ well^−1^) 24 hours before transfection. Synthetic miRNA (0.05, 0.5, 5 pmol), psiCHECK-SM construct, and pGL3-control (Promega) were simultaneously transfected using Lipofectamine 2000 (Invitrogen). After 24 hours of cultivation, cells were harvested and (*Renilla* luciferase activity / firefly luciferase activity) was determined using a Dual-Luciferase Reporter Assay System (Promega). pGL3-Control encoding firefly luciferase served as a control for the calculation of relative luciferase activity for miRNA.

### Calculation of miRNA thermodynamic parameters

Melting temperature (Tm) of each miRNA duplex and seed-target duplex were predicted by means of nearest-neighbor model^54^. The formula for the calculation is as follows. 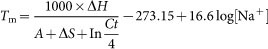


ΔH: Sum of nearest neighbor enthalpy changes (kcal mol^−1^)

A: Helix initiation constant (−10.8 cal mol^−1^ K^−1^)

ΔS: Sum of nearest neighbor entropy changes (kcal mol^−1^ K^−1^)

R: Gas constant (1.987 cal deg^−1^ mol^−1^)

Ct: Total molecular concentration (100 µM)

[Na^+^]: Sodium ion concentration (100 mM)

Nearest-neighbor parameters, enthalpy and entropy, for Watson-Crick base pairing are described by Xia et al.^54^ and those for G:U pairing, by Mathews et al.^55^.

### Statistical analysis

Student t-test was carried out for assessing the correlation between relative luciferase activity and Tm_2–8_, miTm_1–5_ (Tm_1–5_), or Tm_2–8_ − 0.53 × miTm_1–5_ (Tm_1–5_) value, and that between Tm_2–8 _and miTm_1–5_ (Tm_1–5_). Kolmogorov-Smirnov (K-S) test was carried out to validate the difference of significance in Tm values of miRNAs of each species.

## Author Contributions

N.H. and K.U.-T. designed the experiments. N.H. and K.H. carried out the reporter assay experiments. Y.N. analyzed microarray data in the initial stage of this work, and N.H. and E.S. accomplished microarray data analyses. K.U.-T. drafted the manuscript.

## Supplementary Material

Supplementary Information Supplementary Figures

Supplementary Information Supplementary Tables
