# Supplementary material for: Stability of miRNA 5′terminal and seed regions is correlated with experimentally observed miRNA-mediated silencing efficacy
Source: Sci Rep. 2012 Dec 18;2:996. doi: 10.1038/srep00996 (PMC3524778; doi:10.1038/srep00996)

**Supporting information**

Stability of miRNA 5′terminal and seed regions is correlated with experimentally observed miRNA-mediated silencing efficacy

Naoki Hibio^1^, Kimihiro Hino^2^, Eigo Shimizu^2^, Yoshiro Nagata^1^ and Kumiko Ui-Tei^1,2^

**^1^**Department of Computational Biology, Graduate School of Frontier Sciences, University of Tokyo, 5-1-5 Kashiwanoha, Kashiwa-shi, Chiba-ken 277-8561, Japan, **^2^**Department of Biophysics and Biochemistry, Graduate School of Science, University of Tokyo, 7-3-1 Hongo, Bunkyo-ku, Tokyo 113-0033, Japan.


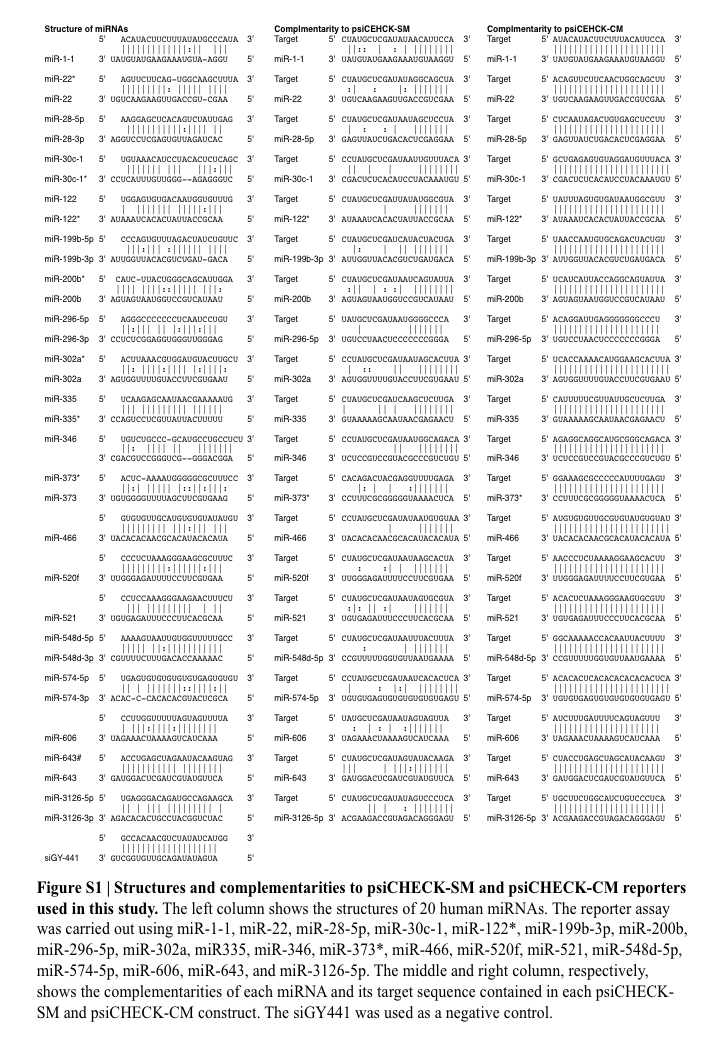


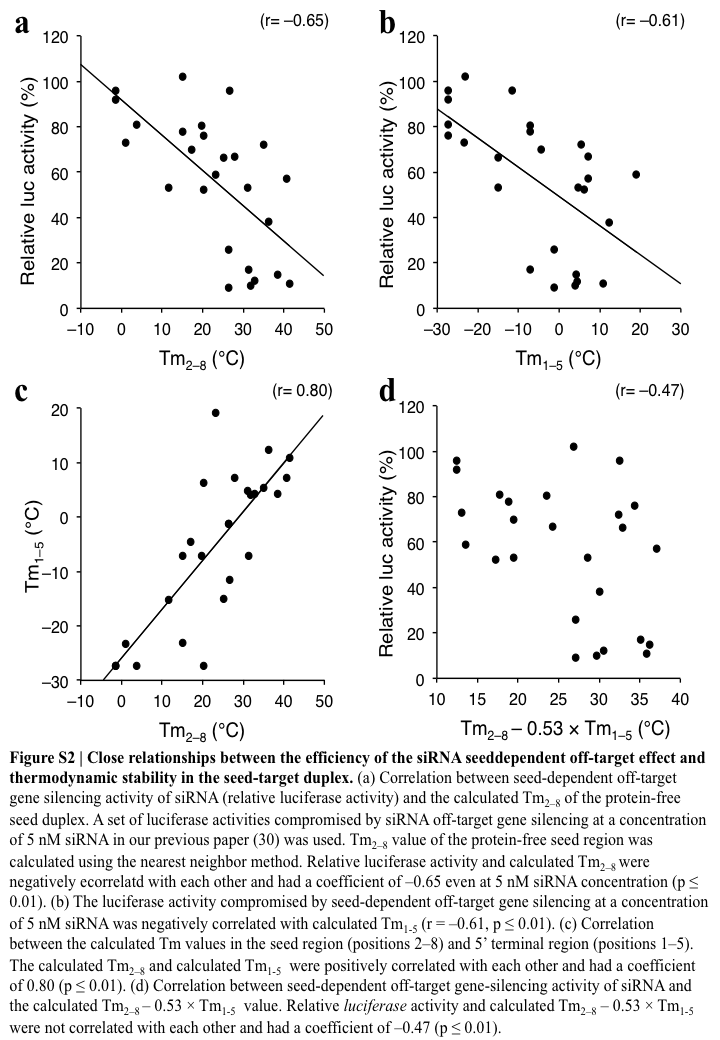


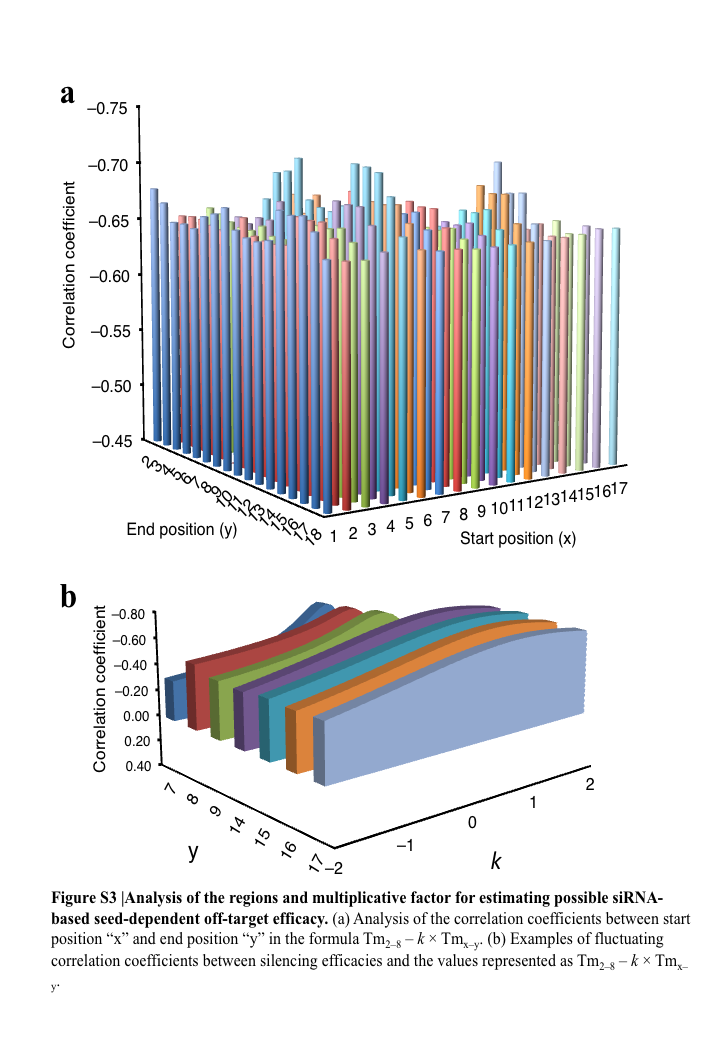

Supplement: Supplementary Information — Supplementary Figures [file srep00996-s1.doc]
